# Correction

**DOI:** 10.1080/20018525.2026.2728252

**Published:** 2026-09-15

**Article title**: Effects of long-term, home-based, virtual cycling after COPD exacerbation – results from the randomised controlled COPDtoParis trial

**Authors**: Anna L. Stoustrup, Lars P. Thomsen, Jane Andreasen, Thorvaldur S. Palsson, Anne Vingaard Olesen, Kristina K. Christensen, Dennis Grønhøj and Ulla M. Weinreich

**Journal**: *European Clinical Respiratory Journal*

**Bibliometrics**: Volume 13, Number 01, pages 1-15

**DOI**: https://doi.org/10.1080/20018525.2026.2673711

It has been noted by the authors that few values in Table 3 were incorrect in the published article. The corrected version of Table 3 has been placed below.

**Table 3.** Outcomes at baseline and 12 months

**Outcome**	Group (n)	Baseline [95 % CI]	12 months [95 % CI]	Mean differences between groups	p-value
**5 RSTS (seconds)**	Intervention (18)	18.3 [13.8;22.7]	11.8 [6.5;17.0]	-1.1 [-8.1;6.0]	0.8
	Control (19)	12.0 [7.6;16.5]	12.8 [8.1;17.5]		
**30s STS (repetitions)**	Intervention (21)	10.5 [0.1;20.9]	11.7 [-0.1;23.4]	-3.5 [-18.2;11.2]	0.6
	Control (19)	13.2 [-0.1;26.6]	15.2 [-0.2;30.6]		
**Sedentary time (hours)**	Intervention (21)	21.6 [20.5;22.7]	21.5 [19.5;23.4]	2.3 [-0.2;4.8]	0.08
	Control (18)	19.2 [17.9;20.4]	19.2 [17.6;20.7]		
**Lung function, FEV1% pred.**	Intervention (19)	44.1 [38.7;49.6]	44.7 [38.5;50.9]	0.2 [-8.3;8.6]	0.9
	Control (19)	46.3 [40.8;51.8]	44.6 [38.8;50.4]		
**SPPB, points**	Intervention (21)	5.1 [3.7;6.5]	5.3 [3.5;7.1]	-1.3 [-3.8;1.1]	0.3
	Control (19)	6.4 [4.9;7.8]	6.6 [5.0;8.3]		
**SGRQ, points**	Intervention (18)	54.9 [47.5;62.3]	49.2 [40.8;57.6]	-3.2 [-14.6;8.2]	0.6
	Control (19)	57.4 [50.1;64.8]	52.4 [44.6;60.1]		
**CAT, points**	Intervention (18)	11.3 [8.0;14.6]	10.6 [6.7;14.4]	-4.7 [-9.9;0.5]	0.08
	Control (19)	13.6 [10.4;16.9]	15.2 [11.7;18.7]		
**EQ-5D-5L, index score**	Intervention (18)	0.8 [0.6;0.9]	0.6 [0.5;0.8]	-0.1 [-0.3;0.2]	0.6
	Control (19)	0.7 [0.6;0.8]	0.7 [0.5;0.8]		
**EQ-VAS, 0-100**	Intervention (18)	61.9 [51.8;72.1]	69.3 [57.0;81.7]	23.3 [6.7;39.9]	0.006
	Control (18)	58.3 [48.2;68.5]	46.1 [35.0;57.1]		
**LSA-DK, points**	Intervention (18)	58.5 [45.4;71.6]	69.7 [54.9;84.6]	26.9 [6.6;47.2]	0.009
	Control (19)	56.0 [43.3;68.8]	42.8 [29.0;56.6]		

**Abbreviations: Confidence interval (CI), 5RSTS, five-repetition sit-to-stand test; 30s STS, 30-second sit-to-stand test; FEV1% pred., forced expiratory volume in 1 second (% predicted); SPPB, Short Physical Performance Battery; SGRQ, St George’s Respiratory Questionnaire; CAT, COPD Assessment Test; EQ-5D-5L, EuroQol 5-Dimensions 5-Levels; EQ-VAS, EuroQol Visual Analogue Scale; LSA-DK, Life-Space Assessment, Danish version**

In addition, in the Primary outcomes section, the sentence:

“Participants unable to perform the 5-repetition sit-to-stand test were assigned a score of 0.”

should read:

“Participants unable to perform the 5-repetition sit-to-stand test were treated as missing.”

This correction has not changed the description, interpretation, or the original conclusions of the article. The authors apologize for any inconvenience caused.

Updated table:

